# Decision support in psychiatry – a comparison between the diagnostic outcomes using a computerized decision support system versus manual diagnosis

**DOI:** 10.1186/1472-6947-8-9

**Published:** 2008-02-08

**Authors:** Lars G Bergman, Uno GH Fors

**Affiliations:** 1 Dept. of Learning, Informatics, Management and Ethics (LIME), Karolinska Institutet, Berzelius way 3, S- 171 77 Stockholm, Sweden

## Abstract

**Background:**

Correct diagnosis in psychiatry may be improved by novel diagnostic procedures. Computerized Decision Support Systems (CDSS) are suggested to be able to improve diagnostic procedures, but some studies indicate possible problems. Therefore, it could be important to investigate CDSS systems with regard to their feasibility to improve diagnostic procedures as well as to save time.

**Methods:**

This study was undertaken to compare the traditional 'paper and pencil' diagnostic method SCID1 with the computer-aided diagnostic system CB-SCID1 to ascertain processing time and accuracy of diagnoses suggested. 63 clinicians volunteered to participate in the study and to solve two paper-based cases using either a CDSS or manually.

**Results:**

No major difference between paper and pencil and computer-supported diagnosis was found. Where a difference was found it was in favour of paper and pencil. For example, a significantly shorter time was found for paper and pencil for the difficult case, as compared to computer support. A significantly higher number of correct diagnoses were found in the diffilt case for the diagnosis 'Depression' using the paper and pencil method. Although a majority of the clinicians found the computer method supportive and easy to use, it took a longer time and yielded fewer correct diagnoses than with paper and pencil.

**Conclusion:**

This study could not detect any major difference in diagnostic outcome between traditional paper and pencil methods and computer support for psychiatric diagnosis.

Where there were significant differences, traditional paper and pencil methods were better than the tested CDSS and thus we conclude that CDSS for diagnostic procedures may interfere with diagnosis accuracy. A limitation was that most clinicians had not previously used the CDSS system under study. The results of this study, however, confirm that CDSS development for diagnostic purposes in psychiatry has much to deal with before it can be used for routine clinical purposes.

## Background

Correct and efficient diagnostic procedures in medicine are gaining more attention for a number of reasons. For instance, an increased demand for new methods to deal with epidemiology, healthcare management, evidence-based medicine, medical guidelines, cost-effective healthcare and saving time are examples that highlight the need for improved diagnostic procedures; and most important – an accurate diagnosis facilitates appropriate treatment outcomes.

In psychiatry, beside ICD (International Classification of Diseases), methods such as the DSM (Diagnostic and Statistical Manual) are used to improve the patient interview process in order to gain more reliable information to support an effective and accurate diagnosis. The use of physical examination findings and laboratory test results are not always relevant or appropriate in improving the diagnostic procedure in psychiatry as it is in other medical specialties.

Computerized decision support for psychiatry has been proposed as a possible enhancement of diagnostic accuracy and also to save time. Standardized structured psychiatric interviews for making a diagnosis go back to computer programs such as DIAGNO [1]. The use of standardized interview techniques and categorization by computers might yield reliable symptom ratings and precise diagnosis [2]. For example, Structured Clinical Interview for DSM Clinical version (SCID-CV) and a structured computer-based method, Computer Assisted Diagnostic Interview (CADI), were significantly better than the unstructured Traditional Diagnostic Assessment (TDA) according to Miller et al. [3].

SCID (Structured Clinical Interview for DSM) was developed to facilitate DSM diagnosis in psychiatry. SCID might be compared to the NIMH (National Institute of Mental Health) diagnostic interview schedule or the DIS method described by Spitzer [4]. The DIS questions determine whether a symptom has been present or not by answering simple 'yes' or 'no' questions.

SCAN (Schedules for Clinical Assessment in Neuropsychiatry) [5], developed by the WHO [6], is an instrument that uses computer algorithms to make a DSM-IV diagnosis. The DIS and SCAN have much in common in their technical make-up with 'yes' or 'no' answers.

The SCID, in contrast, allows more flexibility and professional evaluation of answers and other information sources [4]. Subjects can describe symptoms in their own words in the SCID interview. The questions are designed to elicit important information and the interviewer makes a judgement about the different DSM criteria.

CB-SCID1 is a computerized decision support system that is designed to support and facilitate axis 1 DSM-IV diagnosis in psychiatry. The system sums up fulfilled criteria and presents noted diagnoses.

However, using a Computerized Decision Support System (CDSS) such as CB-SCID1 may trigger unanticipated errors due to automation bias, that is, users may act as the CDSS directs, regardless of the correctness of the action. Users may miss events because the system did not alert them (errors of omission) and do what the system tells them (errors of commission) [7]. However, social accountability can make users spend more time verifying the correctness of the CDSS suggestions which therefore might lead to fewer errors [8].

In the CB-SCID1, a path is selected in the program for a specific patient by judging each criterion at a time and then the program moves on automatically. However, it is possible to regret a judgement in the course of the program.

According to many developers of CDSS systems, they can save time and enhance diagnostic reasoning; however, the computer system might have difficulties solving problems in a global context and may also have a low capacity to integrate global and sequential processing.

Previous studies on CDSS systems for medical purposes have shown both positive and negative results. In a systematic review on the effects of CDSS [9] the author found that many CDSSs improve practitioner performance. Patient outcomes remain understudied and, when studied, inconsistent results were found. In the Garg et al. study it was noted that several CDSS systems studied were inefficient and required more time and effort from the user compared with paper-based methods [9].

Other potential problems in using CDSS have also been described. For instance, in our earlier study, potential problems were identified as missing the correct diagnosis and over-diagnosing [10].

CDSS may also cause problems such as disagreement with clinicians. For example, poor agreement was found between clinicians' diagnoses and the computerized CIDI-Auto for DSM-IV diagnoses (CIDI = Composite International Diagnostic Interview) [11].

Social phobia, major depressive episode and post-traumatic stress disorder had kappa values below 0.40. A slightly higher kappa value (0.52) was found for obsessive-compulsive disorder. All kappa values were below 0.60 [11].

In a comparison between the self-administered CIDI-Auto computerized diagnosis (ICD-10) and psychiatrist routine clinical diagnosis (ICD-10) agreement was only 56%. Moreover, there were numerous subjects who were given a diagnosis of depression from a psychiatrist but not from the CIDI-Auto [12].

In conclusion, CDSS systems are suggested to be able to improve diagnostic procedures in psychiatry, but some studies indicate possible problems.

### Objectives

It may be important to investigate the fairly new CDSS system CB-SCID1 regarding its feasibility to improve the level of diagnostic success as well as the possibility of saving time. Thus, this study was undertaken to compare the traditional 'paper and pencil' diagnostic method SCID1 with the computer-aided diagnostic system CB-SCID1 with regard to processing time and the accuracy of suggested diagnoses.

## Methods

The time required to make a diagnosis is an important variable in almost any medical domain today and is also the case for psychiatry. Therefore, time was chosen as one of the central variables in this study. Other important outcome variables in this study are correct diagnosis and possible incorrect diagnoses.

The CB-SCID1 automatically notes time and diagnosis within the system. The time for the paper and pencil method was clocked by a chronometer and the diagnoses were obtained from the manual compilation of diagnoses on paper.

The individual experiences of the paper and pencil method and the computer method were mapped in an interview with open-ended questions (pros and cons with the paper and pencil method and computer method, respectively) directly after the trial. The answers were coded into thematic categories and the frequency for each category was recorded.

Two real but unidentified patient cases were picked from the DSM-IV Case Book [13] for use in the study. The cases in the book have been collected from a large number of clinicians (experts in particular areas of diagnoses and treatment). One of our cases was considered to be rather complex with three diagnoses and the other was more simple with only one diagnosis.

The correct diagnoses according to the DSM-IV Case Book [13] are used as the 'gold standard' in this study:

Correct Axis 1 diagnosis for the easy case was:

• Brief Psychotic Disorder with Marked Stressors.

Correct Axis 1 diagnoses for the difficult case were:

• Major Depressive Disorder, recurrent, mild

• Somatization Disorder

• Alcohol Dependence, in sustained full remission

Correct diagnoses or number of diagnoses for the patient cases were not revealed to the subjects.

### Subjects

Sixty-three clinicians volunteered to participate in the study. Thirty were specialists in psychiatry, 24 were clinical psychologists, two were general practitioners (specialists), one was a specialist in clinical neurophysiology, one physician, two were physicians in psychiatric specialist training and three were in clinical psychologist training (last year).

The subjects were instructed to diagnose two patient cases: one 'easy' and one more complex with three different diagnoses ('difficult'). Which case was considered as easy or difficult, nor the number of diagnoses for the different cases, was revealed for the subjects. Both cases were described in text.

The SCID procedures by the different subjects were randomized to prevent order effects for both methods (paper and pencil-based SCID1 versus computer-based CB-SCID1) and order of cases interviewed (easy case versus difficult case). All subjects used both methods and both cases but in different combinations according to the randomized schema (see more information in the section Statistical methods, below).

Structured questions, constructed by the authors, and graded on a four-point scale were given in the pre-assessment survey about computer skill and attitude to computer-aided diagnostics. (See Table 1). The questions were put in a clear statement which they could agree to/not agree to in an ordered categorical scale (Strongly disagree = 1, Disagree = 2, Agree = 3, Strongly agree = 4). The subject areas asked about, computer skill and attitude to computer-aided diagnostics, were well defined and familiar to the users, why standardized attitude scales were not used.

**Table 1:** Pre-assessment survey interview questions

**Variable**	**Question**
Professional experience	How many years have you been working as a psychiatric specialist/licensed psychologist ?
DSM training	How many hours of DSM training do you have ?
SCID training – paper and pencil	How many hours of SCID training, paper and pencil, do you have ?
SCID training – computer	How many hours of SCID training, computer (CB-SCID1), do you have ?
SCIDs carried out – paper and pencil	How many SCIDs, paper and pencil, have you carried out ?
SCIDs carried out – computer	How many SCIDs, computer (CB-SCID1), have you carried out ?
Computer skill (1–4)	I am used to work with computers in my daily work Strongly disagree = 1, Disagree = 2, Agree = 3, Strongly agree = 4
Attitude to computer-aided diagnostics (1–4)	I find it appropriate to work with computer-aided diagnostics Strongly disagree = 1, Disagree = 2, Agree = 3, Strongly agree = 4

In the post-assessment survey open-ended questions about pros and cons for the paper and pencil SCID1 and the computer-administered CB-SCID1, were asked, respectively and spontaneously given answers were recorded.

The follow-up interviews were analysed and thematic categories were built according the content and meaning of the answers. No further questioning was pursued. Some subjects gave answers in many thematic categories and some gave answers in few categories and some did not give any answers at all. As the categories were constructed after the interview from the spontaneously given subjective interview data, they are not comparable to each other in no other way than the absolute frequency for each category that stands for itself and functions as complementary information to the objective log-file data and correlations.

### SCID1

SCID1 (Structured Clinical Interview for DSM-IV axis 1 Disorders) is a paper and pencil instrument to facilitate making axis 1 DSM-IV diagnoses [14,15].

The questions are both structured and, as a complement, freely formulated, which makes this method a semi-structured interview support. A pre-clinical interview is always recommended before starting the SCID1 interview. Structured questions are used to facilitate judgement about the fulfilment of different criteria, yes or no answers. The fulfilment of a certain number of criteria makes a diagnosis, provided the instructions are understood and followed.

SCID1 is not a psychometric instrument *per se *and the result is partly dependent on the user's SCID1 training level and clinical experience.

### CB-SCID1

CB-SCID1 is a decision support system designed to support DSM-IV diagnoses in psychiatry. The system is based on the paper and pencil SCID1, including the DSM rules and decision tree. As it was a rule-based system from the beginning, it was straightforward to computerize it. The CB-SCID1 is considered to be of advantage when it deals with the administration in the program for instance, correction of criteria judgement, summing up of fulfilled criteria according to DSM rules, presentation of noted diagnoses and execution of some diagnostic conflict control [16]. The program is functioning like this: the user is asked to judge whether various criteria, presented one by one, are fulfilled or not and the system chooses how to move on, based on user input. The system guides the user through the various branches in the decision tree, based on the "yes", "no" or "unclear" answers about each criterion. The program forces the user to face all major clinical syndromes but it is always possible the move to the next branch in the decision tree by answering no and users are not forced to go into detail in any specific syndrome area. If the number of fulfilled criteria reaches a certain level (according to DSM-IV) the program automatically suggests the corresponding DSM-IV diagnosis.

### Procedure and instruments

The subjects undertook the paper and pencil SCID1 and the CB-SCID1 computer program after a short instruction given by one of the study leaders about how to use the system. They were allowed to read the written cases for 10 minutes before starting and the procedure was briefly as follows:

• *Step 1 – General information: *Oral and written information to subjects about the study, aim, participation, implementation, ethics, handling of data, secrecy and results.

• *Step 2 – Individual information and questionnaire: *Subjects were asked to fill in a questionnaire about gender, age, professional training, DSM-IV training, paper and pencil SCID training, computer CB-SCID1 training, number of paper and pencil SCID interviews done, number of computer CB-SCID1 interviews done, computer skill and attitude towards computer-aided diagnostics.

• *Step 3 – Paper and pencil SCID1 and CB-SCID1 test: *The subjects received instructions on how to use SCID1 and CB-SCID1 and were asked to start working with the systems, respectively, according to the randomized schema, and to attempt to diagnose the written cases. One of the study leaders answered the subjects' questions about technical issues of the systems in the trial, but not about judgement of criteria.

• *Step 4 – Follow up interview: *A follow-up interview, with open-ended questions, directly after the diagnostic trial about pros and cons for the paper and pencil SCID1 and the computer-administrated CB-SCIDI. The questions were non-directive, and only about the pros and cons, recording spontaneously given answers.

### Statistical methods and analysis

A randomization list for the subjects was generated before the trial. The randomization was carried out on the two conditions (computer/paper and pencil) and 'degree of case difficulty' (easy/difficult) and was applied to the starting combinations in blocks of four subjects.

The four starting combinations were: 1, Easy case on computer; 2, Easy case on paper and pencil; 3, Difficult case on computer; and 4, Difficult case on paper and pencil.

A subject starting with the easy case on the computer got the difficult case on paper and so on.

To examine the distribution of easy and difficult cases within the professional categories, Fisher's exact test was applied. In comparison between subjects solving the case on computer and those solving the case with paper and pencil, the Chi-square test was used for variables measured on a nominal scale. The Mann-Whitney *U *test was used to analyse ordered categorical data or continuous data. Associations between baseline information variables and the outcome measures were analysed by Spearman rank order correlations. P < 0.05 was considered statistically significant.

### Ethical approval

All parts of this study have been approved by the ethical committee of Karolinska Institutet. All individual data remained anonymous.

## Results

### Baseline information variables

Descriptive statistics for baseline information variables are presented in Table 2. Of the 63 clinicians only two had been trained and had performed SCID interviews using the CB-SCID1 system before this trial. These two varied greatly: one general practitioner had received four hours training in computer SCID and had carried out 100 cases and one psychologist also had four hours of training, but had only carried out one case.

**Table 2:** Median and percentile values P_10_, P_25_, P_75_, P_90_, n = 63

**Variable**	**Median**	**P**_**10**_	**P**_**25**_	**P**_**75**_	**P**_**90**_
Professional experience (years)	8	0	2	16	24
DSM training (hours)	8	0	0	12	32
SCID training – paper and pencil (hours)	0	0	0	8	12
SCID training – computer/CB-SCID1 (hours)	0	0	0	0	0
SCIDs carried out – paper and pencil (number)	3	0	0	15	35
SCIDs carried out – computer (number)	0	0	0	0	0
Computer skill (1–4) Strongly disagree = 1, Disagree = 2, Agree = 3, Strongly agree = 4	3	2	3	4	4
Attitude to computer-aided diagnostics (1–4) Strongly disagree = 1, Disagree = 2, Agree = 3, Strongly agree = 4	3	2	3	4	4

In many occasions, the clinicians indicated "0" as their answer: Professional experience 16 % of the clinicians indicated "0", DSM training 25 % = 0, SCID training – paper and pencil 59 % = 0, SCID training – computer (CB-SCID1) 97 % = 0, SCIDs carried out – paper and pencil 43 % = 0; and SCIDs carried out – computer 97 % = 0.

No significant correlation was found between DSM training and diagnostic outcome for the clinicians who solved the easy case using paper and pencil and the difficult case using computer support. However, there was a significant correlation between DSM training and diagnostic outcome for the clinicians who solved the easy case using computer support and the difficult case using paper and pencil concerning total number of diagnoses (r = -0.39) and incorrect diagnoses (r = -0.37) for the difficult case. This means that fewer diagnoses and fewer incorrect diagnoses were produced with more training hours in DSM for the difficult case using paper and pencil.

There were no significant correlations between diagnostic outcome and computer training or attitude to computer-assisted diagnostics.

### Time and diagnoses

Regarding the time and diagnostic success variables for the easy case, descriptive statistics are presented in Table 3 for the paper and pencil versus the computer-supported groups.

**Table 3:** Easy case using paper and pencil vs. easy case using computer support

**Variable**	**Paper and pencil **(median and inter-quartile values P_25_-P_75_)	**Computer support **(median and inter-quartile values P_25_-P_75_)
Total time (seconds)	Median 1086 P_25 _635 P_75 _1595	Median 1168 P_25 _938 P_75 _1507
Tot nr of diagnoses	Median 1	Median 1
Tot nr of correct diagnoses	Median 0	Median 0
Tot nr of incorrect diagnoses	Median 1	Median 1

No significant differences for total time and diagnostic variables were found for paper and pencil processing of the easy case compared with computer processing of the easy case.

Descriptive statistics for time and diagnostic variables for the difficult case using paper and pencil versus the difficult case using computer support are presented in Table 4.

**Table 4:** Difficult case using paper and pencil versus difficult case using computer support

**Variable**	**Paper and pencil **(median and inter-quartile values P_25_-P_75_)	**Computer support **(median and inter-quartile values P_25_-P_75_)
Total time (seconds)	Median 1577 P_25 _828 P_75 _2236	Median 1848 P_25 _1626 P_75 _2263
Tot nr of diagnoses	Median 4	Median 4
Tot nr of correct diagnoses	Median 2	Median 2
Tot nr of incorrect diagnoses	Median 2.5	Median 2

A significant shorter total time was found for paper and pencil processing of the difficult case compared with computer processing of the difficult case (p = 0.04).

Correct diagnosis by type of diagnosis for the difficult case using paper and pencil versus computer support is presented in Table 5.

**Table 5:** Correct diagnosis by type of diagnosis, difficult case, for paper and pencil versus computer support

**Diagnose**	**Paper and pencil (Correct diagnose in per cent)**	**Computer support (Correct diagnose in per cent)**
Alcohol	70	76
Somatization	40	60
Depression	53	21

The correct diagnosis 'Depression' for the difficult case was significantly more often found in the paper and pencil group than in the computer group (p = 0.008). Finding the correct diagnoses 'Alcohol' and 'Somatization' seemed easier using computer support although the differences were not significant. For the easy case there were no differences between the two conditions.

### Follow-up interview

The information (spontaneous unstructured pros and con answers about the paper and pencil and computer system) from the follow-up interview was analysed and structured into thematic categories deduced from the content and meaning of the answers (see Table 6).

**Table 6:** Paper and pencil SCID 1 system and computer-administrated CB-SCID 1 system from deducted categories

**Deducted Categories**	**Pro Paper and pencil **(frequency, absolute numbers)	**Con Paper and pencil **(frequency, absolute numbers)	**Pro Computer **(frequency, absolute numbers)	**Con Computer **(frequency, absolute numbers)
System structure, support	Supportive **5**	Non-supportive **18**	Supportive **40**	Non-supportive **3**
System process, navigation	Easy **5**	Difficult **19**	Easy **13**	Difficult **3**
Systems' administration	Easy	Difficult	Easy **8**	Difficult
Systems' advice, Diagnosis	Heeding	Rejecting	Heeding **1**	Rejecting **15**
System preference	Yes **6**	No	Yes **8**	No
Thinking modality, Global	Easy **18**	Difficult **2**	Easy	Difficult **11**
Thinking modality, step by step	Easy	Difficult	Easy **9**	Difficult **4**
Learning, prepared to learn more about the system	Yes **1**	No	Yes **15**	No **2**
Clinical use, prepared to use the system on real patients	Yes	No	Yes **8**	No **5**

Although a majority (40 out of 63) perceived the CB-SCID1 system to be supportive and easy to use it took the users a longer time and yielded fewer correct diagnoses than the paper and pencil method.

Table 6 also shows that the navigation process for 13 subjects is described as easy using computer support and as difficult for 19 subjects using the paper and pencil. Eighteen subjects find it easy to think globally while working with paper and pencil compared with 11 subjects who find it difficult to think globally in the computer situation. As many as 15 out of 63 rejected the computer system's advice.

Eight subjects seem to be prepared to use the system on real patients.

### Psychiatric specialists and clinical psychologists

The results for the dominant groups of psychiatry, psychiatric specialists and clinical psychologists revealed no significant differences between the computer and paper and pencil situation for the variable correct diagnoses in both the easy and difficult cases. For the difficult case on paper and pencil a significant shorter total time as compared with the difficult case on computer was found (p = 0.03). No significant differences in the comparison between computer-administrated and paper and pencil were found for the number of diagnoses and incorrect diagnoses.

The median value for the total number of diagnosis was 1, and for incorrect diagnosis the median value was also 1 for both the computer-administrated and paper situation in the easy case. The median value for the total number of diagnoses is 5 for the computer and 4 for paper and pencil in the difficult case. Also for the difficult case, the median value for incorrect diagnoses is 3 for the computer and 2.5 for paper and pencil.

When looking at the different correct diagnoses it was found than significantly more users in the paper and pencil situation compared with the computer situation found the correct diagnosis 'Depression' (p = 0.014).

In summary, the three main results are: first, no significant differences were found between computer and paper and pencil, for the easy case; second, a significantly shorter total time for the paper and pencil processing of the difficult case compared with computer processing of the difficult case was found; third, the correct diagnosis 'Depression' in the difficult case was found more often in the paper and pencil processing situation than in the computer processing situation.

## Discussion

In this study where we compared a computerized decision support system with manual diagnosis, no major difference between paper and pencil and computer support was found for the easy case. However, for the difficult case, a difference was found in favour of paper and pencil.

It is hard to make any conclusion for this finding other than that traditional decision making is at least as effective as the computer support tested. The prior training and experience in the different methods, paper and pencil and computer support, were not extensive. However, the lack of training time and experience in the computer method were, to some degree, compensated by the instructions the subjects were given in the actual trial. The clinicians were also supported by one of the study leaders in terms of assistance with handling the program. They were given no assistance in the judgement of the different criteria. On the other hand, working 'backwards' in the program, that is regretting and changing, which is rather complicated in the program, caused problems for almost everyone. These functions of regretting and changing might be easier handled in programs with less complex structure like for instance SCAN. However, such functions might not be used so much because coding patients according to "yes" or "no" answers it not too difficult.

Revisiting and changing earlier decisions from judgments in CB-SCID1 on the other hand, in a decision tree, is more complex. Moreover, CB-SCID1 might have a longer learning curve than more straightforward systems.

A common expectation is that computer support results in faster and easier decisions than those made by paper and pencil. The finding that the paper and pencil method was faster for the difficult case can, to some degree, be explained by the fact that the CB-SCID1 is not wholly automatic. The system demands a thinking process that might be harder for a difficult case (with movements back and forth) during the decision process in the program.

The finding that the correct diagnosis 'Depression' (one of the three diagnoses in the difficult case) was found more often with the paper and pencil method than with the computer support could depend on the thinking and navigation processes. Somehow it might be easier to think globally in the paper and pencil situation than in the step-by-step sequential thinking process necessary in the CB-SCID1.

The discovery that the majority found the CB-SCID1 supportive and easy to use while it takes a longer time and yields fewer correct diagnoses than paper and pencil needs some comment.

The CB-SCID1 seems to lend support to structure in its presentation of the next question according to the DSM decision tree, but makes the processing aspect of global thinking and navigation back and forth in the CB-SCID1 difficult. The program might force the thinking to become sequential and the global thinking become difficult. The thinking process might become fragmented and the navigation process in the program even more difficult.

CB-SCID1 might trigger errors due to automation bias, errors of commission, which is following the direction in the program regardless of the correctness of action, or only applying sequential but no global thinking leading to incorrect diagnoses. Missing the diagnosis 'Depression' in the computer situation may be because of automation bias, errors of omission, or merely applying sequential but no global thinking (cf. [7]). Probably, a free flexible combination of sequential and global thinking, adapted to the demands of the situation, would be more advantageous.

It can also be discussed whether CDSS and paper and pencil methods should be seen as alternative methods or if CDSS should take a complementary role in the ordinary clinical work.

Of course, this is dependent on the degree of automation in the CDSS and type of clinical task to be supported. When comparing these two methods, paper and pencil and computer, it is to some degree a comparison between the brain against the 'intelligence' built in into the CDSS. The human brain is good at global information processing while a computer makes it necessary to follow a logical sequential path and handle one piece of information at a time (in our case decide upon one criterion at a time in the CB-SCID1).

General practitioners in primary care settings have shown interest in the CB-SCID1.

Such a system might be of value to them being responsible for first line help in psychiatric issues in Sweden. The CB-SCID1 might be supportive for general practitioners who often lack the psychiatric domain knowledge.

### Limitations

The use of written text on paper when presenting the cases to be diagnosed has limitations concerning interpretation mode. Some subjects may stick inflexibly to the actual written text. Others may fill in the empty spaces in the text using their clinical experience and imagination. Moreover, there may be frustration at not being able to put follow-up questions, since live patients were not actually used, in order to clarify the picture of the patient. The artificiality of the evaluation conditions, not using real live cases, is a limitation in this study not testing the potential possibility that the software might work better under such conditions.

An other limitation in this study is that just one type of CDSS was studied. CDSS varies in complexity, from categorized information that requires further processing to systems with self-learning capabilities. CB-SCID1 is characterized by deductive inference and automatic generation of diagnosis but requires input judgments for various criteria sequentially presented following a predetermined decision tree in the software.

The fact that very few of the clinicians had tested the CDSS CB-SCID1 before the trial might, of course, influence the outcome of the study. However, very few had also had any type of training in paper and pencil SCID. Furthermore, all subjects were instructed in how to use the CB-SCID1 system before the trial and a CB-SCID1-trained person was available during all sessions to answer any questions regarding the system and its functions.

In this study we defined case complexity as number of diagnoses, easy case had one diagnosis and the difficult case had 3 diagnoses. There are of course other ways of defining case complexity, such as rarity.

Another limitation may be faults in the unit test, for instance, errors in the software and design flaws in software architecture. The design and evaluation processes in CB-SCID1, from requirement analysis to assessment of outcomes, may have some drawbacks. Although the focus of this study is not focused on evaluation of the software part we found some indications of such problems. The follow-up interview revealed, for instance, that at least 15 users rejected the CB-SCID1 diagnostic advice. Some of these rejections may be due to errors in the software, some to unskilled handling of the program or software architecture problems. The type of problems mentioned in the follow-up interview were, for example missing the depressive disorder part, missing the alcohol and other substance-related disorder part, missing brief psychotic disorder, problems with tense in questions, problems with 'over diagnosis'. The CB-SCID1 seems to generate a diagnosis after just one criterion yes-answer, according to some participants in the study, which is for generalized anxiety syndrome and hypochondria.

In summary, the greatest limitation in this study might be the unclear status of CB-SCID1 in terms of the life cycle of information systems. The CB-SCID1 has the status of a mature commercialized product on the market. Yet, one might wonder about the development process from requirement analysis to outcomes assessment. What about architecture design, software programming, unit test and acceptance test ? In this study it is difficult to evaluate the importance of user training and familiarity with CB-SCID1 vis-à-vis probable software problems.

## Conclusion

Despite the possible advantages of computer-aided support for diagnostic processes, this study could not detect any major difference in diagnostic outcome between traditional paper and pencil methods and computer support for psychiatric diagnosis.

Where there were significant differences, traditional paper and pencil methods were better than the CDSS tested.

CDSS for diagnostic purposes may interfere with diagnosis accuracy.

However, a limitation of this study was that most clinicians had not previously used the CDSS system under study.

## Abbreviations

CADI Computer-assisted Diagnostic Interview

CB-SCID 1 Computer-based Structured Clinical Interview for DSM IV – Axis I Disorders

CDSS Computer Decision Support System

CIDI Composite International Diagnostic Interview

CIDI-Auto Computerized Composite International Diagnostic Interview

DIS Diagnostic Interview Schedule

DSM IV Diagnostic and Statistical Manual of Mental Disorders. Fourth Edition

ICD-10 International Classification of Diseases, Tenth version

NIMH National Institute of Mental Health

SCAN Schedules for Clinical Assessment in Neuropsychiatry

SCID Structured Clinical Interview for DSM IV Disorders

SCID 1 Structured Clinical Interview for DSM IV – Axis I Disorders

SCID-CV Structured Clinical Interview for DSM IV Disorders – Clinical version

TDA Traditional Diagnostic Assessment

WHO World Health Organization

## Competing interests

Competing interests exist between the commercial CB-SCID1 system from Pilgrim Press and the SCAN system developed by the World Health Organization. However, the authors have no interests in any of these competitors.

## Authors' contributions

UF has supervised LB in the planning, carrying out and analysis of this research work. LB has carried out all experiments. The manuscript has been written in close collaboration between LB and UF. Both authors have read and approved the final manuscript.

## Pre-publication history

The pre-publication history for this paper can be accessed here:


